# The substrate is not enough: receptor-restricted metabolic targeting of ILC2s in allergic airway disease

**DOI:** 10.1097/IN9.0000000000000094

**Published:** 2026-11-06

**Authors:** Bowen Wang, Vanessa-Vivien Pesold, Omid Akbari

**Affiliations:** 1 Department of Immunology and Immune Therapeutics, Keck School of Medicine, University of Southern California, Los Angeles, CA, USA

**Keywords:** group 2 innate lymphoid cell, regulatory T cells, immunometabolism, metabolic flexibility, programmed cell death protein 1 agonism, airway hyperreactivity

## Abstract

Opposing metabolic perturbations of group 2 innate lymphoid cells (ILC2s) – such as upregulating or downregulating glycolysis and suppressing or preserving oxidative phosphorylation – repeatedly produce the same reduction in airway hyperreactivity. We argue that this does not reflect a contradiction in the literature, but rather a defining property of these cells: ILC2s are tissue-resident sentinels whose metabolism is calibrated by cell-surface receptor expression, local nutrient availability, microenvironmental conditions (such as oxygen tension and pH), and metabolic competition with neighboring immune cells, particularly the regulatory T (Treg) cells that restrain them. Since ILC2 pathogenicity requires the coordinated operation of both the glycolytic and oxidative arms, therapeutic interventions are effective in proportion to how much they restrict their metabolic plasticity rather than which specific pathway they target. Given that ILC2s co-localize with, and are likely to share their nutrient supply with, Treg cells and other neighboring populations, this distinction has major clinical implications; it separates targeted therapies that selectively disarm the effector from nonselective strategies that starve the effector and its suppressor together.

## 1. License to adapt: a paradox at the center of group 2 innate lymphoid cells immunometabolism

Iron is required for pulmonary group 2 innate lymphoid cell (ILC2) effector function. Activated murine and human ILC2s (mILC2s and hILC2s, respectively) upregulate transferrin receptor 1 (TfR1), and iron chelation reduces oxidative phosphorylation (OXPHOS), induces a hypoxia-inducible factor 1-alpha (HIF-1α)-driven glycolytic shift, and attenuates airway hyperreactivity (AHR) in murine models. Additionally, TfR1 expression on ILC2s from patients with asthma correlates with disease severity ^[1]^.

Paradoxically, glycolysis is also widely established as a requirement for pulmonary ILC2 effector function. Activation of ILC2s upregulates HIF-1α and glycolytic flux and blocking the HIF-1α/glycolysis axis reduces ILC2 metabolism, cytokine output, and allergic airway inflammation ^[2]^.

Taken together, these studies indicate that pushing ILC2s both toward and away from glycolysis reduces AHR. Both findings are robust and were obtained in allergen- and interleukin (IL)-33-driven models ^[1,2]^. The conventional response is to attribute such a discrepancy to differences in model, timing, or experimental design.

We propose that resolving this conflict requires relinquishing an assumption inherited from conventional immunometabolic frameworks: that metabolic pathways operate in a binary manner, with one arm pathogenic and the other protective. For certain cell types such as ILC2s, their pathogenicity relies on their metabolic plasticity and therapeutic strategies that restrict this plasticity are protective.

## 2. Never say rigid: metabolism calibrated to the niche

Rather than switching between glycolysis and fatty acid oxidation (FAO), ILC2s engage these pathways simultaneously to meet distinct metabolic demands in parallel. For example, hILC2s utilize OXPHOS to support proliferation and survival and glycolysis to drive cytokine production ^[3]^. Upon activation, ILC2s simultaneously increase glycolytic flux and FAO. Glucose uptake is gated by glucose transporter 1 (GLUT1), which drives glycolytic flux upon IL-33-mediated activation and is itself under programmed cell death protein 1 (PD-1) control, linking nutrient transport directly to receptor signaling ^[3,4]^. ILC2s also acquire exogenous fatty acids via the fatty acid translocase CD36 and store them transiently in lipid droplets through diacylglycerol *O*-acyltransferase 1 activity. These transient lipid depots are under the control of mechanistic target of rapamycin and peroxisome proliferator-activated receptor gamma (PPARγ) before mobilizing them for β-oxidation or phospholipid synthesis ^[5–8]^. Mobilized fatty acids are not restricted to β-oxidation and phospholipid synthesis. They can be re-esterified into neutral lipids, elongated, desaturated, or diverted into the synthesis of signaling lipids such as eicosanoids and sphingolipids ^[9]^. Which metabolic route predominates in ILC2s, and whether this balance shifts with activation state or tissue microenvironment, remains unknown. Notably, PPARγ is required for IL-33-mediated activation of ILC2s in both lung and adipose tissue ^[5]^, indicating that the same regulatory axis must adapt to tissue contexts that differ in lipid composition and availability.

This metabolic flexibility is essential to ILC2 survival and is continuously calibrated by nutrient availability. When the supply of glucose and other micronutrients is constrained, such as during malnutrition or helminth infection, ILC2s rely more heavily on exogenous fatty acids to sustain barrier integrity ^[10]^. Concurrently, amino acid availability sets the magnitude of their response independently of either glycolysis or OXPHOS ^[11]^. Iron availability provides a further layer: experimental iron chelation forces a HIF-1α-driven glycolytic shift, illustrating how depletion of a single nutrient triggers pathway switching rather than uniform shutdown ^[1]^. While these pathways are distinct, they are not isolated: disruption of Arginase-1 (Arg1) inhibits both l-arginine-derived polyamine biosynthesis and the glycolytic machinery. Thus, Arg1 acts as a checkpoint on the whole system rather than as a component of 1 pathway ^[12]^.

However, metabolic flexibility does not necessarily imply metabolic indecision. Rather, it represents the essential evolutionary adaptation of a tissue-resident sentinel, enabling ILC2s to rapidly respond to alarmins across microenvironments that vary widely in oxygen tension and in lipid, glucose, and amino acid availability ^[9,13]^. ILC2s occupy anatomically distinct niches, from the lung to adipose tissue to the tumor microenvironment, and the metabolic program is calibrated to the local nutrient supply rather than hardwired in the cell itself. While nutrient landscapes differ by tissue, we emphasize that direct measurements of interstitial nutrient concentrations in the airway do not, to our knowledge, exist. Consequently, the assumptions that glucose, fatty acids, and amino acids are usually at or near systemic concentrations in healthy airway tissue and that allergic inflammation raises local demand without a commensurate fall in supply both remain untested working hypotheses. The tumor microenvironment is the one setting in which competition for a shared substrate has actually been demonstrated: there, glucose consumption by tumor cells depletes the extracellular pool and starves glycolysis-dependent effector cells ^[14]^. Tissue-specific cytokine milieus further shape ILC2 identity and metabolic adaptation, with IL-6 and tumor necrosis factor-alpha (TNF-α) contributing to tissue-specific ILC2 phenotypes ^[15]^. Taken together, these observations indicate that forcing ILC2s onto a single metabolic track, regardless of which track, is functionally destructive (Figure 1A). The studies discussed in the previous section are consistent with this interpretation: both arrived at the same functional conclusion while disagreeing about mechanism.

**Figure 1.**
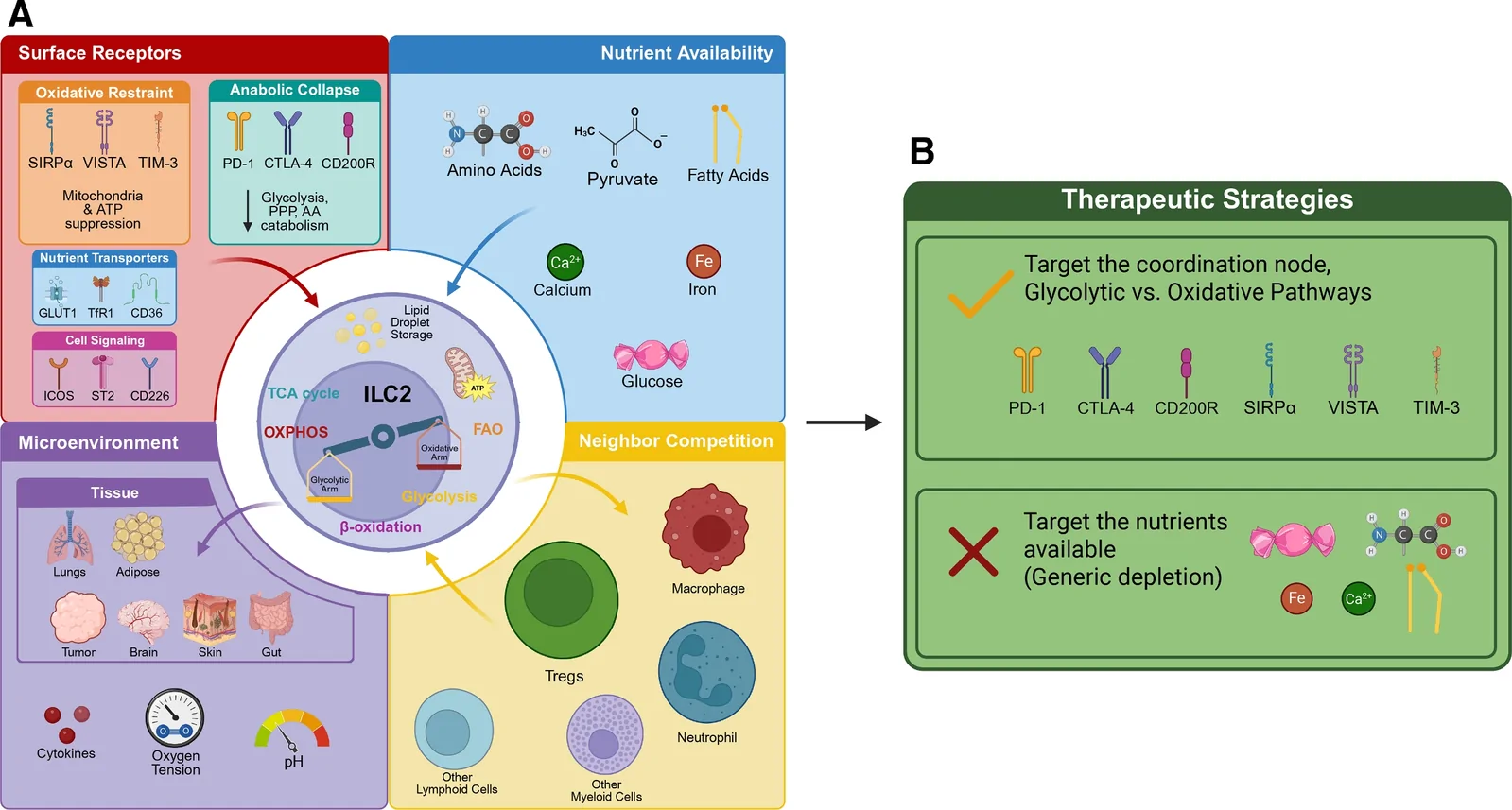
**Therapeutic strategies should target ILC2 metabolic regulation.** (A) ILC2 metabolism is shaped by the expression of activating, inhibitory, and nutrient-sensing surface receptors. Nutrient availability supports major metabolic pathways such as glycolysis, the TCA cycle, OXPHOS, FAO, and lipid-droplet formation. In addition, tissue-specific microenvironmental factors influence ILC2 metabolic activity. Competition for nutrients with other immune cells further determines ILC2 activation, function, and adaptation within inflamed tissues. (B) Potential therapeutic strategies should target the node to disarm the effector cell’s metabolic plasticity. Strategies should avoid depleting the shared substrate pool, which also taxes the suppressor cells occupying the same niche. FAO, fatty acid oxidation; ILC2, group 2 innate lymphoid cell; OXPHOS, oxidative phosphorylation; SIRPα, signal-regulatory protein alpha; TCA, tricarboxylic acid; Tim-3, T cell immunoglobulin and mucin-domain containing-3; Treg: regulatory T cells; VISTA, V-domain immunoglobulin suppressor of T cell activation.

However, one overarching caveat applies to these studies as a whole and should be stated outright. Almost all the metabolic evidence summarized here relies on models that remove ILC2s from their native microenvironment. Fully validating the model proposed here will require moving beyond the perturbation of cultured cells and integrating spatial metabolomics, transcriptomics, and proteomics. This will allow the construction of a spatially resolved metabolic atlas of the allergic lung, which does not yet exist. Generating one is, in our view, the most urgent empirical need in this area.

## 3. Convergence royale: divergent mechanisms, one outcome

When multiple inhibitory receptors are compared, distinct signaling pathways are found to target distinct metabolic machinery, yet the functional outcomes are essentially identical ^[16–18]^. Three receptors, signal-regulatory protein alpha (SIRPα), V-domain immunoglobulin suppressor of T cell activation (VISTA), and T cell immunoglobulin and mucin-domain containing-3 (Tim-3) all suppress the oxidative arm, but through different routes. SIRPα activation suppresses mitochondrial metabolism via the Janus kinase/signal transducers and activators of transcription and extracellular signal-regulated kinase/mitogen-activated protein kinase (MAPK) pathways, whereas its deficiency upregulates both tricarboxylic acid (TCA) cycle activity and OXPHOS. Thus, therapeutic targeting of this regulatory axis alleviates AHR ^[16]^. VISTA activates forkhead box protein O1 (FOXO1) signaling, which restrains ILC2 function, whereas VISTA deficiency increases mitochondrial metabolism ^[17]^. Similarly, Tim-3 agonism acts via Nemo-like kinase to downregulate nuclear factor kappa-light-chain-enhancer of activated B cell (NF-кB) activity and suppresses mitochondrial function and adenosine triphosphate (ATP) production in pulmonary ILC2s to attenuate AHR in both IL-33- and Alternaria-driven models, whereas ILC2-specific Tim-3 deletion exacerbates disease ^[18]^.

There are also other receptors that simultaneously act in opposing directions. Cytotoxic T-lymphocyte-associated protein-4 (CTLA-4) limits activation while preserving mitochondrial respiratory capacity during chronic inflammation ^[19]^. While both CD200R and T cell immunoreceptor with immunoglobulin and immunoreceptor tyrosine-based inhibitory motif domains (TIGIT) engagement attenuates ILC2 function, they do so by distinct mechanisms. CD200R restricts overall ILC2 function to reduce AHR, while TIGIT activation induces ILC2 cell death to limit tissue inflammation ^[20,21]^. Finally, CD226 blockade is the most informative outlier: it upregulates glycolysis while suppressing FAO and lipid uptake, yet still alleviates AHR ^[22]^.

The 7 receptors mentioned above yield at least 3 distinct metabolic signatures – oxidative suppression, glycolytic restraint with preserved respiration, and reciprocal arm swapping. However, they all converge on one consistent phenotype: reduced ILC2-driven airway disease. When such divergent metabolic mechanisms converge on an identical phenotype, the field is describing a principle rather than a series of isolated anomalies. There is, however, one receptor pathway that has tested this metabolic flexibility hypothesis more rigorously than any other, because its signaling cascade has been experimentally pushed in both directions.

## 4. The niche who loved me: PD-1 agonism, blockade, and a second cell in the airway

PD-1 is expressed on naïve and activated mILC2s and hILC2s and is upregulated by IL-33 ^[4,9,13]^. In PD-1-deficient mice, ILC2s exhibit increased glycolysis, pentose phosphate pathway (PPP) activity, and amino acid catabolism while exhibiting lower OXPHOS, which results in ILC2 expansion and, consequently, greater AHR and pulmonary eosinophilia. Conversely, PD-1 agonist treatment restrains ILC2 activity and ameliorates AHR ^[4]^. This establishes a therapeutic strategy in allergic asthma that is inverted relative to oncology: airway protection requires activating an inhibitory signal rather than removing one.

Importantly, PD-1 agonism does not simply induce a global metabolic shutdown in ILC2s; rather, it selectively suppresses specific anabolic pathways such as glycolysis, PPP activity, glutaminolysis, and methionine catabolism, while leaving OXPHOS relatively spared ^[4,9]^. This leaves the cell energetically stable and able to respire, but unable to expand. This mechanism predicts a therapeutic ceiling: PD-1 agonism should be most effective during acute expansion where anabolic demand peaks, and least effective against established, oxidatively maintained tissue-resident populations. PD-1 expression is cell intrinsically gated by liver kinase B1 (LKB1), which maintains mitochondria activity and OXPHOS. Impaired LKB1 activity promotes mitochondrial dysfunction and elevated levels of reactive oxygen species (ROS) ^[23]^. This effectively places PD-1 inside the loop it regulates.

Additionally, 2 observations further complicate the role PD-1 plays in metabolism. First, while PD-1 agonism suppresses airway inflammation, PD-1 blockade on tumor-resident ILC2s promotes TNF-α production and restricts metastatic melanoma progression ^[24]^. In the tumor microenvironment, macrophage-derived signals further regulate PD-1 and CTLA-4 expression on ILC2s, shaping the metabolic state the receptor encounters ^[19]^. This demonstrates that in different tissues, the same receptor requires opposing therapeutic strategies, because the local nutrient environment determines how its signal is metabolically interpreted rather than the receptor dictating a fixed metabolic outcome. Second, the same pathway that restrains ILC2s simultaneously governs suppressor networks. Programmed death-ligand 2 (PD-L2) deficiency impairs airway tolerance to ovalbumin by limiting peripherally induced Treg (pTreg) cell number and function. PD-L2-deficient pTregs show reduced forkhead box P3 (Foxp3) expression, increased methylation of the Treg-specific demethylated region, and impaired TCA cycle activity and ATP production. However, supplying exogenous pyruvate can bypass this mitochondrial blockage and partially restore IL-10 production and airway tolerance in vivo ^[25]^.

Within the lungs, engagement of the same receptor on different cell types produces opposing phenotypes. Engagement of PD-1 collapses anabolic metabolism in the effector and sustains mitochondrial metabolism in the suppressor. This demonstrates that PD-1 is not a receptor with an intrinsic metabolic direction. Rather, its functional readout is determined by the cell type and the metabolic state of that cell’s microenvironment, including oxygen tension, pH, and lactate, which differ fundamentally between healthy, inflamed, and tumor tissue. Hypoxia induces adrenomedullin in lung epithelia, stimulating ILC2 activation and type 2 inflammation, while HIF-1α drives the glycolytic shift supporting ILC2 effector function ^[2,26]^. In highly glycolytic tumors, lactate promotes PD-1 expression on Tregs, creating a feedback loop where the metabolic byproduct of one cell reinforces the suppressive phenotype of another – a loop absent in healthy or acutely inflamed airway tissue where glucose is not yet depleted ^[27]^.

## 5. License to spare: the niche is shared, and so is the fuel

The metabolic limitations of the suppressive arm are fundamental, not incidental. Tregs and ILC2s are not isolated populations that merely occupy the same niche. Both are found within the peribronchial and perivascular compartments and are therefore likely to draw on an overlapping pool of glucose, fatty acids, and amino acids – the same locally variable substrate pool that calibrates ILC2 metabolism in the first place. We stress that this shared access is inferred from co-localization rather than demonstrated by direct measurement of substrate flux in situ, and that establishing it directly is a prerequisite for the therapeutic argument developed below. Furthermore, these 2 cell types have a direct connection. Treg-mediated suppression of ILC2s, which attenuates AHR, requires inducible T-cell costimulator (ICOS)–ICOS ligand interaction and cytokine signaling ^[28]^.

This direct cellular coupling creates 2 complications for potential therapies. First, it imposes a hard constraint on any substrate-targeted therapy. Any therapy that restricts a shared nutrient in the airway microenvironment is not only disarming the effector; it is simultaneously taxing the regulatory cell whose function is to restrain that effector. The PD-L2 study from the previous section makes this risk clear – immune suppression fails when the pTreg TCA cycle collapses ^[25]^. Studies reporting improved AHR after broad metabolic restriction rarely assess Foxp3 stability, risking an unforeseen cost.

Second, the conduit between the 2 cells is intrinsically metabolic. ILC2s express both ICOS and ICOS-L, where ICOS signaling functions within ILC2s as a negative regulator of sterol regulatory element-binding protein 2 (SREBP2)-driven cholesterol biosynthesis and intracrine cortisol production. ICOS–ICOS-L interaction in cis on ILC2s promotes ILC2 function and limits their IL-10 output ^[29]^. Contact-dependent suppression and lipid metabolism are therefore coupled: engagement of a surface receptor can directly reconfigure intracellular lipid handling.

This shared niche also means ILC2s and Tregs compete for fuel, and the outcome depends on the tissue. In the tumor microenvironment, this dynamic inverts and glucose depletion by tumor cells starves glycolysis-dependent effector cells, while Tregs adapt by actively importing lactic acid via monocarboxylate transporter 1, supporting their suppressive function where glucose is scarce ^[14,30]^. This gives Tregs a metabolic advantage over the very cells they suppress, and raises the possibility, which remains untested in the airway, that Tregs preempt locally available nutrients in a way that further constrains ILC2 function. In healthy or inflamed airway tissue, where glucose is thought not to be depleted and lactate to be minimal, this competitive inversion may be less pronounced. The metabolic balance between effector and suppressor is therefore not fixed but depends on which tissue the 2 cells share and what fuel that tissue provides.

Our framework requires 3 crucial qualifications. First, ILC2s and Tregs may not compete for fuel symmetrically: it is not established that their substrate requirements are identical, nor that Tregs are as metabolically flexible as ILC2s appear to be, and Treg suppressive function is comparatively dependent on oxidative and lipid metabolism. Occupancy of a shared niche therefore need not imply symmetrical vulnerability to substrate restriction ^[3,10,25,30]^. Second, ILC2s reside in a complex, IL-33-rich adventitial and perivascular niche alongside T helper 2 (Th2) cells, dendritic cells, macrophages, and nonhematopoietic populations such as stromal and epithelial cells, all of which both consume and supply metabolites as well as signaling cues that shape ILC2 identity and metabolic adaptation ^[15,19,31–34]^. This environment is a multicellular metabolic ecosystem, not just a 2-cell competition, and any complete model of ILC2 metabolic flexibility will have to account for this. Third, niche interactions extend beyond competition. Neighboring cells may cross-feed ILC2s with lactate, fatty acids, or amino acids, while metabolites such as lactate can shape checkpoint expression on neighboring lymphocytes, and sterols and their derivatives act through nuclear and surface receptors to set cellular responsiveness ^[6,7,11,27,29,35]^. Ultimately, competition, cooperation, and metabolic signaling are likely to operate simultaneously. This is a complex dynamic that spatially resolved metabolomics is uniquely equipped to disentangle.

This is why selectivity, rather than the identity of the substrate, is the operative therapeutic variable: restriction of a shared substrate is inherently nonselective across a shared niche, whereas a receptor-restricted intervention can be confined to the effector. Therefore, potential effects on other receptor-expressing immune cells should be carefully evaluated when developing ILC2-targeted therapies.

## 6. No time to stress: ion and nutrient perturbations as the cleanest test

Calcium provides the decisive case for this model. Deletion of Orai1 and Orai2 does not merely redirect ILC2 metabolism; it also collapses glycolysis, FAO, and OXPHOS, resulting in the loss of ILC2 effector function and AHR with no remaining compensatory pathways ^[36]^. By contrast, Piezo1-dependent Ca^2^^+^ influx restrains ILC2s by dampening mitochondrial activity through Krüppel-like factor 2-mediated suppression of NF-κB–a partial constraint producing a partial phenotype ^[37]^.

Disruption of core homeostatic machinery follows the same pattern. Autophagy-related 5 deletion shifts ILC2s toward glycolysis at the expense of FAO and TCA activity, thus impairing cytokine production ^[38]^. Loss of the immunoproteasome i-20S disrupts mitochondrial capacity and abrogates IL-33-induced airway inflammation ^[39]^. Iron, which is essential to ILC2 activation, also exposes it to iron-dependent oxidative stress. To survive this toxicity, activated ILC2s rely on ferroptosis tolerance via glutathione peroxidase 4 and thioredoxin reductase 1 to sustain lipid handling, where the loss of these enzymes prevents ILC2 accumulation ^[40]^. Similarly, von Hippel-Lindau deficiency enhances HIF-1α-dependent glycolysis yet impairs tissue-resident ILC2 maturation by attenuating expression of the IL-33 receptor, suppression of tumorigenicity 2 (ST2), again reinforcing the pattern in which increased glycolysis accompanies compromised function ^[41]^.

Across iron, calcium, autophagy, proteostasis, and redox tolerance, these interventions do not share a pathway. They do, however, share a consequence: the cell can no longer coordinate both metabolic arms. Only one class of lipid departs from this pattern, and that exception is informative.

## 7. The lipid with the golden scaffold: cholesterol on a different tier

Cholesterol clarifies the coordinating rule of ILC2 immunometabolism by being the exception: it serves not as a fuel but as a signaling scaffold. The relevant evidence comes from other cell types. In *Helicobacter pylori*-infected gastric epithelial cells, ST2 and IL-1 receptor accessory protein assemble into cholesterol-rich lipid rafts, and disruption of raft integrity attenuates downstream NF-κB and MAPK signaling without altering receptor abundance. To apply this concept on a broader scale, studies in model membranes and transformed cell lines established raft composition as a determinant of receptor clustering and signal strength ^[42,43]^. Since neither study examined ILC2s, whether ST2 raft residency similarly tunes IL-33 responsiveness in ILC2s remains, to our knowledge, untested. On the strength of the data currently available, we hypothesize, rather than assert as established fact, that membrane lipid composition regulates signaling gain upstream of any decision about substrate use. We regard this as a hypothesis well worth addressing directly. This is consistent with a regulatory role rather than a bioenergetic one: ICOS inhibits SREBP2-driven cholesterol biosynthesis and intracrine cortisol production ^[29]^. The resulting IL-10-producing state requires Blimp-1 and c-Maf, while carrying its own metabolic signature ^[44]^.

Sterol metabolism therefore belongs on a distinct tier apart from FAO and glycolysis: it governs how acutely the cell senses IL-33, rather than what it consumes downstream. Conflating the 2 tiers likely underlies the contradictions in Section 1. This distinction also highlights the dual function of ICOS, which serves as the conduit for Treg-mediated suppression and as an intrinsic sterol checkpoint within ILC2s. Whether other inhibitory receptors discussed here are subject to the same membrane-level control remains untested. While raft residency and palmitoylation are established for programmed death-ligand 1 (PD-L1), comparable studies have yet to evaluate PD-1, SIRPα, or Tim-3 in ILC2s ^[45]^.

## 8. A View to a cure: predictions and the selectivity test

If metabolic flexibility rather than a hardwired reliance on a single fuel constitutes ILC2s’ true vulnerability, 5 testable predictions follow.

First, protection magnitude should track the breadth of metabolic constraint rather than its direction. Orai1/2 deletion should outperform any single-arm intervention ^[36]^. Additionally, the CD226 phenotype, in which one arm rises as the other falls, should yield a milder effect than either–with the caveat that CD226 blockade also lowers overall oxidative metabolism, making it a partial constraint rather than a pure reciprocal swap ^[22]^. This is a mechanistic benchmark, not a therapeutic proposal: Orai channels are required across immune and nonimmune lineages, and that distinction is the subject of the fourth prediction. This warrants systematic comparison across published AHR datasets.

Second, the effects of a dual glycolytic and oxidative inhibitor at individually subtherapeutic doses should be synergistic rather than additive. A fuel-preference model predicts functional redundancy; a flexibility model predicts metabolic collapse ^[3]^. If correct, this is also the first selectivity argument in the framework: doses too low to constrain a resting cell should still collapse one already running both arms at capacity.

Third, PD-1 agonism should exhibit reduced efficacy where ILC2 persistence is oxidatively maintained rather than anabolically driven, with chronic, steroid-refractory disease serving as the benchmark model ^[1,4]^. If agonism functions equally well against acute and established populations, the anabolic-collapse account is invalid.

Fourth, the therapeutic window of any metabolic intervention should be set by how confined its engagement is, not by how deeply it constrains the target cell. The prediction is a contrast between 2 classes of intervention. An unconfined intervention–substrate withdrawal, or a node inhibitor given without a cell-restricted handle–should disable ILC2s and the Tregs beside them together, so that any fall in AHR is accompanied by falling pTreg frequency, loss of Foxp3 stability, and reduced IL-10 output measured in the same animal ^[25]^. A receptor-confined intervention should dissociate the 2, lowering ILC2 effector function while leaving pTreg number and suppressive capacity intact or improved; PD-1 engagement, which collapses anabolic metabolism in the effector while sustaining mitochondrial metabolism in the suppressor, is the first case to test ^[4]^. The same contrast should hold systemically, in compartments that share the pathway but not the disease–thymus, skeletal muscle, gut epithelium, and hematopoietic progenitors ^[1,25,30,41]^. An intervention that lowers AHR at the cost of Treg function, or that registers where there is no disease to treat, has not achieved selectivity; it has relocated the cost.

Fifth, the informative readout is dynamic switching capacity, not steady-state flux. Traditionally, metabolic profiling has relied on single time-point measurements of extracellular acidification and oxygen consumption. Real-time paired measurement of these parameters under substrate challenge would test switching capacity directly, which single-time-point profiling cannot ^[2,3,36,38,40]^. However, such assays still require cells to be removed from the tissue, so they should be paired with spatially resolved measurements. Given the current technological state, we can pair spatial metabolomics with spatial transcriptomics and proteomics from adjacent serial sections to report the metabolic state of an ILC2 together with the identity, position, and metabolic state of its neighboring cells with which it shares substrate. This combination would serve as the definitive test of the framework proposed here.

Our model states that pathogenic ILC2 effector function is driven by its ability to switch between glycolytic and oxidative pathways depending on its local environment. However, this model fails if a perturbation is found that restricts activated ILC2s onto a single metabolic pathway (glycolytic or oxidative), while leaving the other pathway intact and inducible, while still abolishing AHR. To date, no such experimental or clinical data have been reported.

This flexibility model directly addresses a critical clinical problem. In the United States, approximately 26.5 million people, or 8% of the population, are affected by asthma ^[46,47]^. Additionally, of the 26.5 million people with asthma, roughly 30% of these patients fail to achieve sustained disease control on existing therapies, including biologics directed at downstream type 2 cytokines ^[7,46]^. Surface markers–PD-1, ICOS, CTLA-4, and the other inhibitory receptors discussed here–are demonstrably critical gatekeepers of ILC2 metabolic function ^[4,9,19,29]^. Yet receptors represent 1 layer of control rather than the complete picture. They operate within a context set by local nutrient availability, microenvironmental conditions including oxygen tension, pH and cytokine milieu, and competition with co-localized cells such as Tregs ^[2,11,28,30]^.

Inhibiting mitochondrial complexes or FAO is already achievable; what is not is doing so in one cell type, or in one tissue, without imposing the same constraint on every other cell that depends on the same machinery. That, rather than the identity of the pathway, is the problem the field must solve, because the coordination nodes on which ILC2 flexibility depends are general-purpose machinery shared across the host.

Cell-surface receptors offer a way past this obstacle because they supply an “address” rather than a mechanism. Receptor engagement confines the metabolic consequence to cells displaying the receptor and restricted to the activation state and tissue in which they display it ^[9]^. The ideal therapeutic unit is therefore a pairing–a receptor that confers selectivity and a metabolic node that confers effect – with local airway delivery available as a second, orthogonal axis ^[9,20,28]^. PD-1 agonism therefore represents the first proof of concept of that pairing rather than an anomaly. It collapses anabolic metabolism in pulmonary ILC2s while the same pathways keep running in cells that do not display the receptor ^[4,19]^. Judged this way, the measure of a candidate therapy is not how completely it constrains ILC2 metabolism, but how little of that constraint the host is asked to absorb (Figure 1b).

## Conflicts of interest

The authors declare that they have no conflicts of interest.

## Funding

This work was supported by National Institutes of Health Public Health Service grants R01 HL144790, R01 HL151493, R01 AI181866, R01 AI169687, R01 HL151769, and R01 HL159804 (O.A.).
